# Dissecting cellobiose metabolic pathway and its application in biorefinery through consolidated bioprocessing in *Myceliophthora thermophila*

**DOI:** 10.1186/s40694-019-0083-8

**Published:** 2019-11-13

**Authors:** Jingen Li, Shuying Gu, Zhen Zhao, Bingchen Chen, Qian Liu, Tao Sun, Wenliang Sun, Chaoguang Tian

**Affiliations:** Key Laboratory of Systems Microbial Biotechnology, Tianjin Institute of Industrial Biotechnology, Chinese Academy of Sciences, Tianjin, 300308 China

**Keywords:** *Myceliophthora thermophila*, Cellulose, Cellobiose, Malic acid, Metabolic engineering, *Thermothelomyces thermophilus*

## Abstract

**Background:**

Lignocellulosic biomass has long been recognized as a potential sustainable source for industrial applications. The costs associated with conversion of plant biomass to fermentable sugar represent a significant barrier to the production of cost-competitive biochemicals. Consolidated bioprocessing (CBP) is considered a potential breakthrough for achieving cost-efficient production of biomass-based fuels and commodity chemicals. During the degradation of cellulose, cellobiose (major end-product of cellulase activity) is catabolized by hydrolytic and phosphorolytic pathways in cellulolytic organisms. However, the details of the two intracellular cellobiose metabolism pathways in cellulolytic fungi remain to be uncovered.

**Results:**

Using the engineered malic acid production fungal strain JG207, we demonstrated that the hydrolytic pathway by β-glucosidase and the phosphorolytic pathway by phosphorylase are both used for intracellular cellobiose metabolism in *Myceliophthora thermophila*, and the yield of malic acid can benefit from the energy advantages of phosphorolytic cleavage. There were obvious differences in regulation of the two cellobiose catabolic pathways depending on whether *M. thermophila* JG207 was grown on cellobiose or Avicel. Disruption of *Mtcpp* in strain JG207 led to decreased production of malic acid under cellobiose conditions, while expression levels of all three intracellular β-glucosidase genes were significantly up-regulated to rescue the impairment of the phosphorolytic pathway under Avicel conditions. When the flux of the hydrolytic pathway was reduced, we found that β-glucosidase encoded by *bgl1* was the dominant enzyme in the hydrolytic pathway and deletion of *bgl1* resulted in significant enhancement of protein secretion but reduction of malate production. Combining comprehensive manipulation of both cellobiose utilization pathways and enhancement of cellobiose uptake by overexpression of a cellobiose transporter, the final strain JG412*Δbgl2Δbgl3* produced up to 101.2 g/L and 77.4 g/L malic acid from cellobiose and Avicel, respectively, which corresponded to respective yields of 1.35 g/g and 1.03 g/g, representing significant improvement over the starting strain JG207.

**Conclusions:**

This is the first report of detailed investigation of intracellular cellobiose catabolism in cellulolytic fungus *M. thermophila*. These results provide insights that can be applied to industrial fungi for production of biofuels and biochemicals from cellobiose and cellulose.

## Background

Lignocellulosic biomass has long been recognized as a potential sustainable source for many industrial applications, including the biosynthesis of biofuels and commodity chemicals. Once established, these processes would make important contributions to rural development and enhanced sustainability of agricultural landscapes. The processes of microbial conversion of plant cell walls include cellulolytic enzyme production, saccharification of plant biomass, and synthesis of the desired products [1, 2]. The costs involved in conversion of insoluble plant lignocellulose into fermentable sugar remain a significant barrier to commercialization. Economic analysis has revealed that consolidated bioprocessing (CBP), which implies incorporating cellulase secretion and the biochemical biosynthetic pathway into a single cell, will enjoy the benefit of cost-efficient production of biomass-based fuels and commodity chemicals [3, 4]. Recently, cellulolytic organisms, such as *Trichoderma*, *Neurospora*, *Clostridium*, and *Myceliophthora* have been considered as the CBP strain candidates for producing biochemicals directly from plant cell walls [5–8].

When cellulolytic organisms grow on plant cell wall, extracellular endo-/exo-glucanases synergistically depolymerize cellulose with cellobiose as the major product [9]. After being imported into the cell by a cellodextrin transporter, cellobiose is mainly cleaved through two pathways; namely, the hydrolytic pathway and the phosphorolytic pathway. In the hydrolytic pathway, β-glucosidase converts cellobiose to two molecules of glucose, which can be further metabolized through glycolysis, while the phosphorolytic pathway uses cellobiose phosphorylase to cleave intracellular cellobiose with inorganic phosphate (Pi) to produce one glucose molecule and one glucose-1-phosphate molecule. Glucose-1-phosphate is then catalyzed to glucose-6-phosphate by phosphoglucomutase, without the need for ATP. Thus, the phosphorolytic pathway requires one ATP for each molecule of cellobiose to be metabolized by glycolysis, while two ATP molecules are consumed for phosphorylation of glucose generated by hydrolysis of cellobiose to form glucose-6-phosphate in the first step of glycolysis [10, 11]. In organisms, the energy advantages of phosphorolytic cleavage would provide extra ATP for microbial growth, cellulase synthesis, and even production of biochemicals from plant cellulose. The phosphorolytic pathway together with cellobiose transport have been incorporated into *Saccharomyces cerevisiae* for improved ethanol production from cellobiose [12, 13]. There is increasing evidence that a relative dominance of phosphorolytic cleavage over hydrolytic intracellular cleavage of cellobiose is widespread in cellulolytic anaerobic bacteria; for example, in *Ruminococcus albus*, *Prevotella ruminicola*, and *Clostridium thermocellum* [10, 14, 15]. Nevertheless, the detail of the two intracellular cellobiose utilization pathways in aerobic cellulolytic fungi remains to be investigated.

The thermophilic filamentous fungus *Myceliophthora thermophila* (Synonym: *Thermothelomyces thermophilus*) is able to secret a large amount of hydrolytic enzymes and grow robustly on cellulosic materials, making it exceptionally attractive for biorefinery application [16, 17]. *Myceliophthora thermophila* has been developed into a mature system for carbohydrate hydrolase production at industrial level (C1 strain) [18]. The multiple characteristics mentioned above and the capability to assimilate all sugar released from plant biomass qualify this fungus as the promising CBP strain candidate [19]. Recently, we incorporated the export system of malic acid and elevated metabolic flux of the reductive tricarboxylic acid (rTCA) pathway in *M. thermophila*. The resultant strain was able to produce malic acid by direct conversion of hemicellulose and cellulose [7]. Of four native synthetic pathways to malic acid, the rTCA pathway is considered the most simple and efficient. The process starts with the carboxylation of pyruvate (from glycolysis) to oxaloacetate and then subsequent reduction to l-malic acid [20, 21]. Theoretically, the pathway can fix 1 mol CO_2_/1 mol malate and processes the highest theoretical yield of 2 mol/mol glucose with redox balance. However, the energy balance for malic acid synthesis via the rTCA pathway is barely even, because modest ATP needs to support both microbial growth and cellulase synthesis. Therefore, the yield of malic acid should benefit from increased intracellular ATP concentration. In this study, using the previously engineered malic acid production strain JG207 of *M. thermophila*, we investigated the behaviors of the phosphorolytic and hydrolytic cellobiose catabolic pathways for application to malic acid production from cellobiose and Avicel. Through combined engineering of the two cellobiose utilization pathways and enhanced cellobiose uptake, malic acid production by *M. thermophila* showed significant improvement when grown on cellobiose or Avicel. These results provide novel insights that can be applied to industrial fungi engineering for the production of bio-based fuels and chemicals from plant biomass.

## Results

### Investigation of intracellular cellobiose metabolic pathway in *M. thermophila*

In cellulolytic filamentous fungi, the cleavage of intracellular cellobiose into glucose is mainly catalyzed via the hydrolytic pathway [22, 23]. *Myceliophthora thermophila* genome encodes at least eight genes encoding predicted β-glucosidase enzymes, including four secreted β-glucosidases and four intracellular enzymes. However, a previous systems-level transcriptomic study indicated that only three intracellular members (*bgl1*, Mycth_115968; *bgl2*, Mycth_38200; and *bgl3*, Mycth_62925) showed significant increased transcription levels during growth on Avicel or plant biomass [17, 24]. Although three secreted β-glucosidases were identified by mass spectrometry in the supernatant of *M. thermophila* culture grown on plant biomass [25], their expression levels were extremely low [17, 24]. Based on transcriptomic data, we hypothesized that the three intracellular β-glucosidase genes (*bgl1*, *bgl2*, and *bgl3*) might be the most relevant enzymes in the cellobiose hydrolytic pathway of *M. thermophila*.

**Fig. 1:**
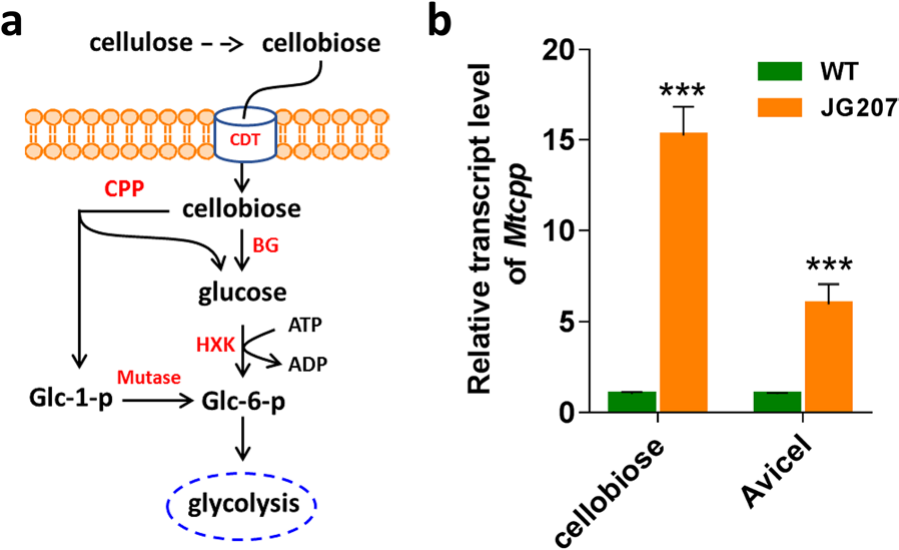
Intracellular metabolic pathway of cellobiose. **a** Overview of intracellular cellobiose cleavage. **b** Transcriptional levels of *Mtcpp* in malate-producing strain *Myceliophthora thermophila* JG207 grown on cellobiose and Avicel for 2 days. *CPP* cellobiose phosphorylase, *BG* β-glucosidase, *CDT* cellodextrin transporter, *HXK* hexose kinase

In the cellobiose phosphorolytic pathway, there is only one predicted cellobiose phosphorylase encoding by gene Mycth_2308030 (named as *Mtcpp* here) in the *M. thermophila* genome, which was classified into Glycoside Hydrolase family 94 (GH94). Previously, one malate-producing strain JG207 of *M. thermophila*, which exhibited fast utilization of variable carbon sources including cellobiose and Avicel, was constructed through overexpressing malate transporter gene (*mae*) and pyruvate carboxylase gene (*pyc*) in the wild-type *M. thermophila* [7]. The transcriptional level of *Mtcpp* was found to be significantly increased in strain JG207 under cellobiose or Avicel conditions, compared with that in the wild-type *M. thermophila* strain via RT-qPCR analysis (Fig. 1b). This suggested the predicted MtCPP is of physiological relevance and might play a crucial role in the efficient metabolism of intracellular cellobiose. Based on these data, we predicted that both cellobiose cleavage pathways exist and are involved in intracellular cellobiose catabolism in *M. thermophila* (Fig. 1a).

### Disruption of *Mtcpp* dramatically affects malate production in *M. thermophila*

**Fig. 2:**
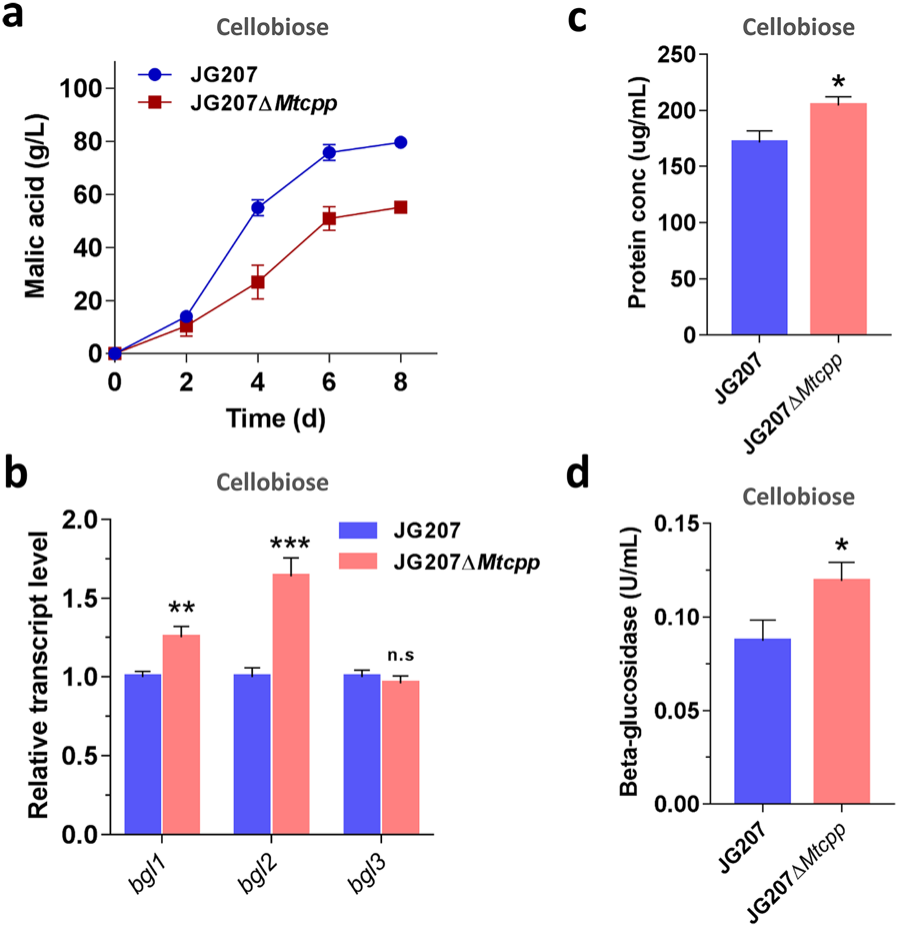
Phenotypes of the strain JG207*ΔMtcpp* on cellobiose in shaking flask. **a** Profiles of malic acid production. **b** Transcriptional levels of intracellular β-glucosidase genes (*bgl1*, *bgl2*, and *bgl3*) in strain JG207*ΔMtcpp* grown on cellobiose for 2 days. The protein concentration (**c**) and activity of β-glucosidase (**d**) in the supernatant of JG207*ΔMtcpp* culture after 4 days on cellobiose. The values and error bars represent means and standard deviations of independent triplicate experiments, respectively

To assess the role of the phosphorolytic pathway on intracellular cellobiose utilization when *M. thermophila* is grown on cellobiose and cellulose, we created the cellobiose phosphorylase gene (*Mtcpp*) deletion strain on the background of the JG207 strain. The *Mtcpp* gene in the *M. thermophila* JG207 strain was disrupted via homologous replacement with a *neo*-inclusive cassette mediated by the CRISPR/Cas9 system [26]. The correct recombination events in the resultant mutants were confirmed by PCR analysis (Additional file 2: Figure S1). When grown on 75 g/L cellobiose, the resultant JG207Δ*Mtcpp* strain produced 55.2 g/L malic acid, representing a 30.7% decrease in the malate titer when compared with the parental JG207 strain (79.7 g/L) (Fig. 2a). In contrast, protein secretion, extracellular β-glucosidase activity and biomass of the JG207Δ*Mtcpp* strain were higher than in the parent strain; protein concentration, β-glucosidase activity and biomass were increased by 19.3%, 36.7% and 15%, respectively (Fig. 2c, d and Additional file 3: Figure S1). A previous report indicated that cellobiose and its derivatives can function as an inducer of lignocellulolytic enzyme gene expression in cellulolytic fungi [23]. The increased protein secretion might result from reduced efficiency of intracellular cellobiose degradation generated by the deletion of *Mtcpp*. Although a significantly increased transcriptional level of *bgl2* was detected via RT-qPCR analysis (Fig. 2b), the deficiency of cellobiose utilization in disruption of *Mtcpp* was not rescued by an alternative cellobiose hydrolysis pathway enhanced by up-regulating β-glucosidase. This clearly suggests that the phosphorolytic pathway is the dominant pathway for cellobiose utilization in this thermophilic fungus when grown on cellobiose.

**Fig. 3:**
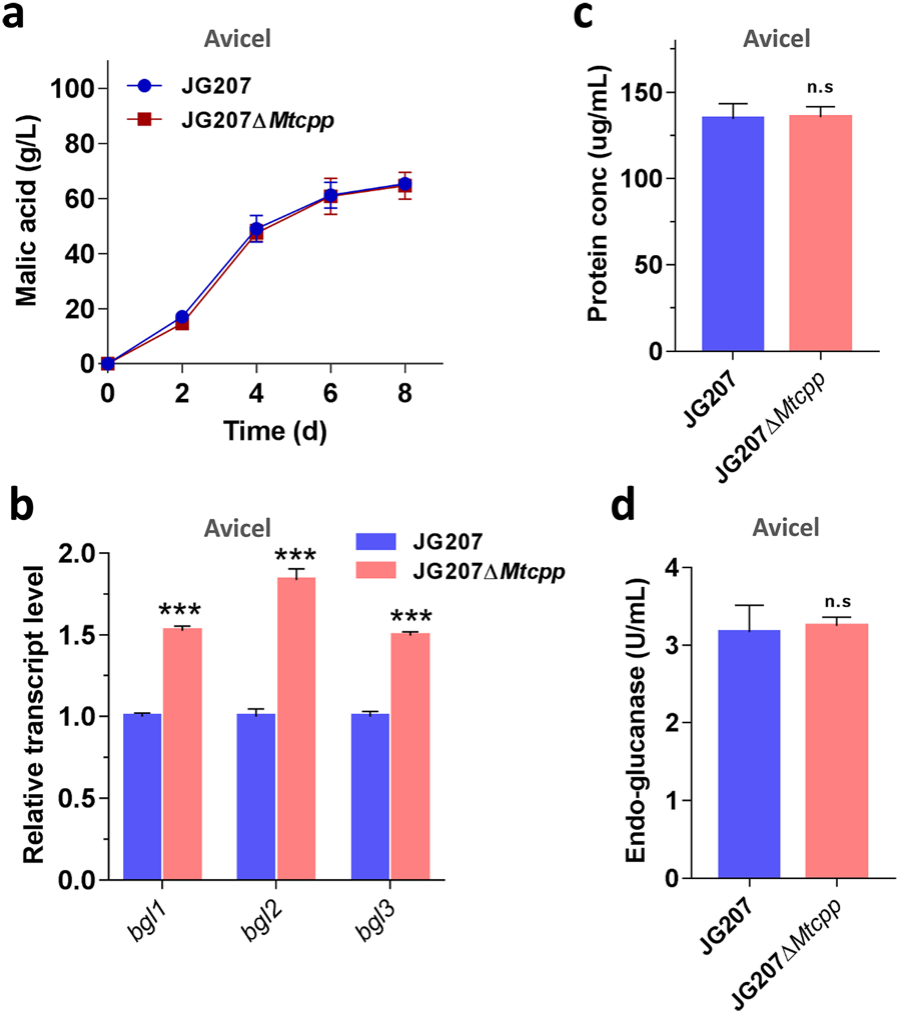
Physiological characterization of the strain carrying the deletion of *Mtcpp* on Avicel in shaking flask. **a** Time-course of malic acid production. **b** Transcriptional levels of intracellular β-glucosidase genes (*bgl1*, *bgl2*, and *bgl3*) in strain JG207*ΔMtcpp* grown on Avicel for 2 days. The protein concentration (**c**) and activity of β-glucosidase (**d**) in the supernatant of JG207*ΔMtcpp* culture after 4 days on Avicel. The values and error bars represent means and standard deviations of independent triplicate experiments, respectively

When grown on Avicel, surprisingly, no significant change was observed on production of malic acid by the JG207Δ*Mtcpp* strain when compared with that of the JG207 parent strain (Fig. 3a). In addition, the biomass, protein secretion and enzyme activities of JG207Δ*Mtcpp* culture were similar to those of strain JG207 (Fig. 3c, d and Additional file 3: Figure S1). Deletion of *Mtcpp* resulted in significantly elevated transcriptional levels of all three intracellular β-glucosidase genes, which might rescue the impairment of the disrupted phosphorolytic pathway on cellobiose metabolism (Fig. 3c). These results indicated that: (1) different regulation patterns of intracellular cellobiose degradation may emerge depending on whether *M. thermophila* is grown on cellobiose or cellulose, (2) the hydrolysis pathway of cellobiose might play a bigger role when *M. thermophila* is grown on Avicel.

### Enhanced cellobiose uptake facilitates faster cellobiose fermentation and malate production

Despite the energy advantage of the phosphorolytic pathway, the rate of cellobiose phosphorolysis was limited by the unfavorable energetics of the reaction (*ΔG*° = + 3.6 kJ mol^−1^) [27]. Rapid substrate supply was recognized as one strategy to maintain a high flux of reaction [28]. Moreover, fast uptake of substrate is a prerequisite for efficient cell factory production of biochemicals [25]. Cellobiose transporter gene *cdt*-*1* from *N. crassa* has been systematically characterized and used for improving uptake of cellobiose [29]. Therefore, for more efficient uptake of cellobiose into the host, the genes *cdt*-*1* was fused to the strong constitutive promoter of *eif* (encoding elongation initial factor) and incorporated into the *M. thermophila* JG207 strain. After confirmation of the presence of the transgene by PCR analysis (Additional file 2: Figure S1), the physiological characterizations of resultant strain JG207cdt was conducted when grown on cellobiose and cellulose.

**Fig. 4:**
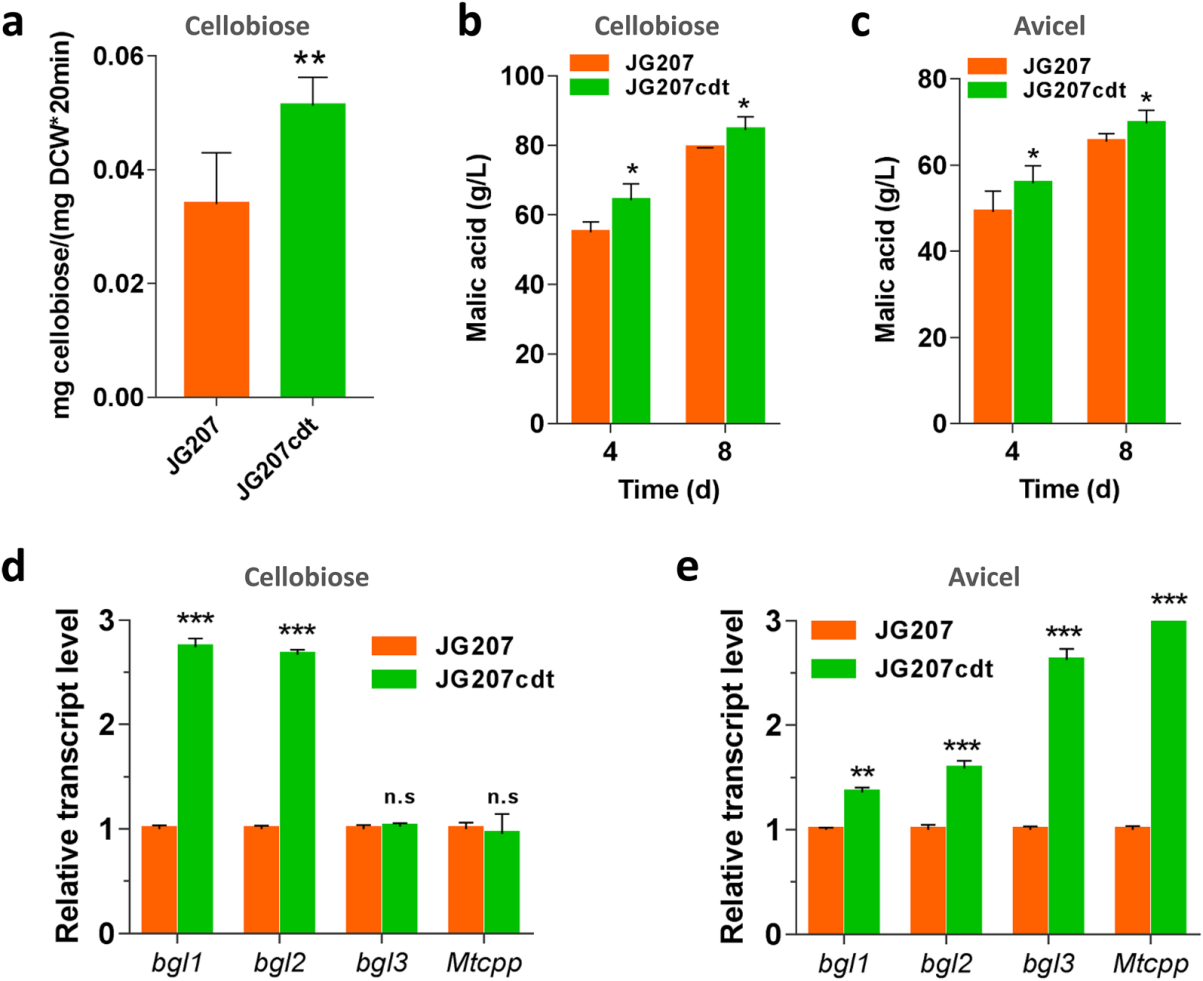
The acceleration of cellobiose uptake. **a** Cellobiose transport rate of the mycelia from the strain JG207cdt overexpressing cellobiose transporter gene *cdt*-*1* from *Neurospora crassa*. Titers of malic acid in the culture of JG207cdt were determined when grown on cellobiose (**b**) and Avicel (**c**) after 4 days and 8 days in shaking flash. **d**, **e** Transcriptional levels of intracellular β-glucosidase genes and cellobiose phosphorylase gene in strain JG207cdt when grown on cellobiose and Avicel for 2 days

When compared with the parent strain JG207, the uptake rate of cellobiose was improved by 51% in strain JG207cdt with 6 copies of *cdt*-*1* in its genome (Fig. 4a and Additional file 4: Figure S1). When grown on cellobiose and Avicel for 4 days, strain JG207cdt produced 64.3 g/L and 55.8 g/L malic acid, representing 17% and 14% increases in the malate titer (55.0 g/L on cellobiose and 49.1 g/L on Avicel), respectively. After 7 days of culture, malic acid titers were 84.4 g/L and 69.7 g/L on cellobiose and Avicel, respectively, corresponding to yields of 1.13 g/g and 0.93 g/g, respectively (Fig. 4b, c). These results indicate that enhancement of cellobiose uptake led to increased production of biochemicals from plant biomass. Subsequently, transcriptional levels of the genes involved in intracellular cellobiose degradation were analyzed when substrate uptake was elevated. When compared with the parent strain JG207, the genes *bgl1* and *bgl2* encoding β-glucosidase exhibited significantly increased expression levels in strain JG207cdt under cellobiose conditions (Fig. 4d), while the transcription levels of all three intracellular β-glucosidase genes and cellobiose phosphorylase gene *Mtcpp* were significantly improved in strain JG207cdt when grown on Avicel (Fig. 4e). These results clearly show that to improve the performance of the cell as a factory for producing bio-based chemicals under both cellobiose and cellulose conditions, engineering the two intracellular cellobiose metabolic pathways simultaneously would be a good strategy.

### Elevation of metabolic flux of phosphorolytic pathway is propitious to efficient production of malic acid from cellobiose

**Fig. 5:**
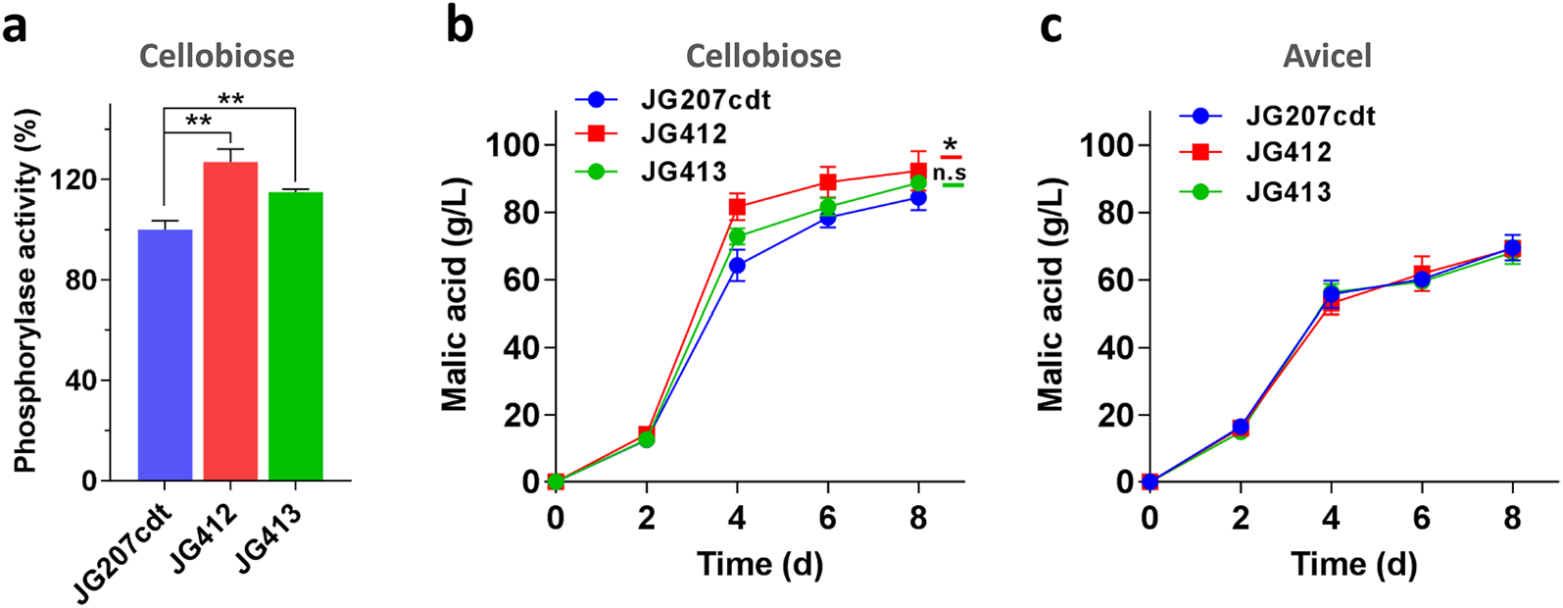
Elevation of metabolic flux of phosphorolytic pathway by overexpressing cellobiose phosphorylase gene. Each of cellobiose phosphorylase genes (*Ctcpp* or *Mtcpp*) was co-incorporated with cellobiose transporter gene *cdt*-*1* into *M. thermophila* JG207 to generate strains JG412 and JG413, respectively. **a** The activity of cellobiose phosphorylase in strain JG412 and JG413 relative to strain JG207cdt after 2 days of shaken-flask culture on cellobiose. Fermentation profiles of engineered *M. thermophila* with cellobiose phosphorylases on cellobiose (**b**) and Avicel (**c**) in shaking flask. Values are the mean of two independent fermentations and error bars represent the standard deviation

Given the results above, overexpression of phosphorolytic enzyme-encoding gene was performed to improve the cell factory chemical production ability through enhancement of phosphorolytic pathway efficiency. In previous report, the gene *Ctcpp* from themophilic bacterium *C. thermocellum* has been characterized and functionally overexpressed in *S. cerevisiae* to improve ethanol production from cellobiose [28]. Therefore, companied with the *cdt*-*1*-overexpressing cassette, each of two cellobiose phosphorylase genes *Mtcpp* and *Ctcpp* behind strong constitutive promoter of *eif* were introduced into strain JG207 to generate the JG412 and JG413 strains, respectively. RT-qPCR analysis indicated that 4 copies of *Ctcpp* and 5 copies of *Mtcpp* were integrated into the genomes of the strains JG412 and JG413, repectively (Additional file 4: Figure S1). As expected, the activities of phosphorylase were increased by 15% and 27% in strains JG412 and JG413, respectively, when compared with the parental strain (Fig. 5a). These data suggest that the *Ctcpp* gene from anaerobic bacteria can be functionally expressed in thermophilic filamentous fungi. When grown on cellobiose, the JG412 and JG413 strains consistently produced 92.4 g/L and 88.84 g/L, respectively, 9.5% and 5.2% more than that produced by strain JG207cdt overexpressing cellobiose transporter gene *cdt*-*1* (Fig. 5b). This indicates that the yield of malic acid can benefit from the energy advantages of phosphorolytic cleavage of cellobiose. However, as shown in Fig. 5c, extra overexpressing cellobiose phosphorylase gene had no effect on malate production under Avicel conditions, which is consistent with the hypothesis above that the hydrolytic pathway plays the major role for cellobiose utilization under cellulose conditions.

### Malate production further improved by simultaneous engineering of two cellobiose catabolism pathways

**Fig. 6:**
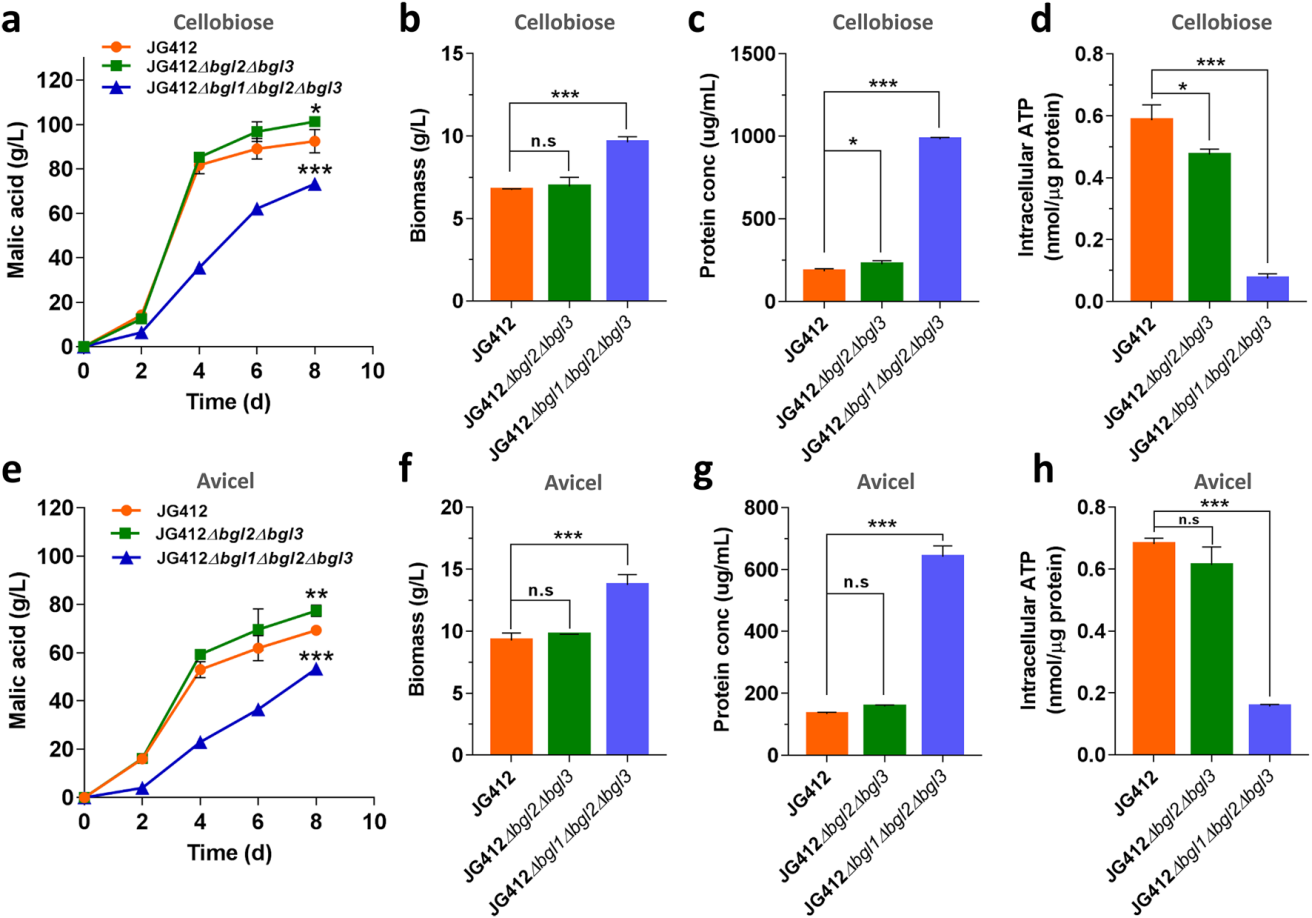
Effect of the distinct combination of intracellular β-glucosidase gene deletion sets on production of malic acid. Physiological characterizations of the strains JG412*Δbgl2Δbgl3* and JG412*Δbgl1Δbgl2Δbgl3* were determined when grown on cellobiose (**a**–**c**) and Avicel (**d**–**f**) in shaking flask. **a**, **e** Time-course of malate production. **b**, **f** Dry weights of mycelia in the culture of these strains grown on cellobiose and Avicel for 8 days. **c**, **g** Protein concentration in the supernatant after 4 days of culture. **d**, **h** Assay of ATP concentration in strains grown on cellobiose and Avicel for 2 days, respectively

To further tune the fungal cell factory to produce malic acid from cellobiose and cellulose, we sought to simultaneously engineer the two cellobiose catabolic pathways. Importantly, we knew there is only one phosphorylase gene in this thermophilic fungus, whereas there are three β-glucosidase genes that might be involved in the cellobiose hydrolytic pathway. In cellulolytic filamentous fungi, the expression levels of genes encoding β-glucosidase were much higher than those of phosphorylase genes when responding to cellobiose and even plant biomass [17, 23, 30], while deletion of all three main β-glucosidase genes led to defective cellobiose utilization [22, 31]. To further investigate the effect of β-glucosidase gene disruption in *M. thermophila* on production of malic acid from cellobiose and Avicel, seven mutant strains, including single, double, and triple mutant strains carrying different combinations of glucosidase gene deletion sets on the background of strain JG412, were constructed and tested. When grown on cellobiose or Avicel, four mutant strains (JG412*Δbgl1*, JG412*Δbgl1Δbgl2*, JG412*Δbgl1Δbgl3*, and JG412*Δbgl1Δbgl2Δbgl3*) with the deletion of *bgl1* showed significantly decreased the titer of malic acid. In particular, strain JG412*Δbgl1Δbgl2Δbgl3* produced the lowest malate titers (58.6 g/L and 52.0 g/L, corresponding to 6.1 g/g biomass and 3.9 g/g biomass), which were 34% and 23% less than parental strain JG412 (69.3 g/L and 92.4 g/L, corresponding to 13.7 g/g biomass and 7.5 g/g biomass) on cellobiose and Avicel, respectively (Fig. 6a, e), although final biomass was improved by 42.5% and 48.2% on cellobiose and Avicel, respectively. Two individual β-glucosidase deletion strains (JG412*Δbgl2* and JG412*Δbgl3*) produced similar titers of malic acid and biomass compared with strain JG412. Only strain JG412*Δbgl2Δbgl3* exhibited similar biomass production but gave an increased titer of malic acid when grown on cellobiose and Avicel. After growth on cellobiose and Avicel for 8 days, titers of malic acid reached 101.2 g/L and 77.4 g/L (corresponding to 14.6 g/g biomass and 8.0 g/g biomass, respectively), with respective yields of 1.35 g/g and 1.03 g/g (Fig. 6a, b and d, f). These results clearly suggest that the ability of the fungal cell factory to produce biochemicals can be further improved by fine regulation of two cellobiose metabolic pathways, combined with elevated uptake of substrate.

In *N. crassa* and *Penicillium decumbens*, deletion of all main β-glucosidase genes that provide the bulk of glucose-generating activity from cellobiose led to impaired cellobiose degradation and enhancement of protein secretion [23, 32]. When assessing secreted protein of the mutant strains in response to cellobiose or Avicel, we found that four mutant strains (JG412*Δbgl1*, JG412*Δbgl1Δbgl2*, JG412*Δbgl1Δbgl3*, and JG412*Δbgl1Δbgl2Δbgl3*) displayed significantly increased protein secretion. The highest level of secreted protein was achieved in the culture of strain JG412*Δbgl1Δbgl2Δbgl3* when grown on cellobiose and Avicel. These data indicated that β-glucosidase encoded by *bgl1* was the dominant enzyme in converting cellobiose to glucose. The highest-level malate producer JG412*Δbgl2Δbgl3* secreted a slightly elevated protein when grown on cellobiose and Avicel, whereas mutant strains JG412*Δbgl2* and JG412*Δbgl3* secreted protein at levels similar to parent strain JG412 (Fig. 6c, g).

Based on the fact that protein synthesis and secretion consume ATP, which is the cofactor of malate dehydrogenase catalyzing the conversion of oxaloacetate to malate, we hypothesized that decreased malate production might result from lower intracellular ATP concentration in mutant strain JG412*Δbgl1Δbgl2Δbgl3*. Therefore, assays of intracellular ATP were performed for the mutants. As shown in Fig. 6, the intracellular ATP concentrations of strain JG412*Δbgl1Δbgl2Δbgl3* were decreased by 87% and 77% relative to parental strain JG412 when grown on cellobiose and Avicel, respectively (Fig. 6d, h).

## Discussion

Cellulosic plant biomass has many desirable features as a potential energy source, but is difficult to efficiently convert into biofuels and commodity chemicals. The approach of CBP represents a promising technology for achieving this conversion in a cost-efficient manner [4]. To achieve low-cost production of bioethanol and biochemicals from cellulose via CBP, there is a real need for improved understanding of the mechanisms relating to intracellular cellobiose, which is the major end product of cellulase.

Previous works have reported that two pathways, the hydrolytic and phosphorolytic pathways, are used to efficiently degrade intracellular cellobiose in cellulolytic organisms. The hydrolytic pathway based on β-glucosidase is relatively widespread and is found in cellulolytic bacteria and filamentous fungi [11]. With bioenergy advantages, the phosphorolytic pathway seems to be limited almost entirely to bacteria and all published examples of cellobiose degradation are from bacteria, especially anaerobic bacteria [10, 33, 34]. The benefits of phosphorolytic cleavage of cellobiose can partly compensate for ATP consumption in supporting microbial growth and cellulase synthesis [11]. In contrast to cellulolytic anaerobic bacteria, cellular respiration in aerobic filamentous fungi can provide sufficient ATP for maintaining cell growth and cellulase production at the expense of NADH generated through catabolism of the carbon source. To date, few reports have emerged on the cellobiose degradation via phosphorylase in filamentous fungi. However, when developed as the CBP host, the energy advantages of phosphorolytic cleavage of cellobiose providing extra ATP in filamentous fungi would be beneficial in treatment of plant biomass. In this study, we found that enhancement of cellobiose catabolic flux led to improved expression levels of phosphorylase gene in *M. thermophila* when grown on cellobiose and Avicel, suggesting that the phosphorolytic pathway worked together with β-glucosidase to catalyze the degradation of cellobiose.

In filamentous fungi, systematic transcriptomic profiles of cellulase genes presented a large divergence in response to cellulose and its hydrolysate cellobiose [23]. Herein, disruption of the phosphorolytic pathway resulted in significantly decreased production of malic acid when *M. thermophila* JG207Δ*Mtcpp* was grown on cellobiose. However, the reduction in malic acid production in strain JG207Δ*Mtcpp* was not significantly different from that observed in the parent strain JG207 in cellulose condition. Transcriptional levels of all three intracellular β-glucosidase genes were significantly elevated to rescue the impairment of the disrupted phosphorolytic pathway on cellobiose metabolism under Avicel conditions. Enhancement of phosphorolytic cleavage by overexpression of the cellobiose phosphorylase gene also led to distinct fluctuations in the production of malic acid in response to cellobiose and Avicel. These trends revealed that in *M. thermophila*, the hydrolytic pathway plays the major role under cellulose conditions, whereas the phosphorolytic pathway contributed more in terms of intracellular cellobiose cleavage under cellobiose conditions.

The ability of a sugar transporter to dominate sugar input into cell factories that link extracellular biomass utilization and intracellular metabolic pathways is considered a critical factor for efficient biosynthesis of desired product in CBP [25, 29]. Engineering a sugar transporter to reduce released sugar was beneficial to relieving inhibition of fungal cellulases by their hydrolysis, for instance cellobiose and cellodextrin [35]. Moreover, the phosphorolysis reaction is unfavorably energetic and high substrate concentration is necessary to maintain an efficient flux of the reaction. In *S. cerevisiae*, cellobiose fermentation by the phosphorolytic pathway was greatly improved by using cellodextrin transporter with elevated rates of cellobiose transport [28]. When grown on cellulose, overexpression of cellobiose transporter gene *cdt*-*1* from *N. crassa* simultaneously enhanced the metabolic flux of the hydrolytic and phosphorolytic pathways. However, when under cellobiose conditions, only β-glucosidase genes showed significantly increased transcriptional levels when uptake of cellobiose was improved. Even so, increased production of malic acid and protein secretion in culture supernatants of the strain overexpressing *cdt*-*1* were detected when grown on cellobiose and Avicel, which is consistent with the previous report that a precise regulation network adjusts cellulase secretion and lignocellulose degradation according to intracellular metabolic efficiency in *M. thermophila* [7].

Previous works reported that cellobiose or a modified version of cellobiose function as an inducer of lignocellulolytic gene expression and when responding to cellobiose or Avicel, the deletion of the main β-glucosidase genes led to efficient induction of cellulase gene expression in filamentous fungi, such as *N. crassa*, *Trichoderma reesei*, and *P. decumbens* [23, 32, 36]. When these mutant strains with divergent combinations of glucosidase gene deletion sets were cultivated on cellobiose or cellulose, we found that the mutants with deletion of the main β-glucosidase gene *bgl1* showed significantly increased protein secretion, which was distinguished from the phenomenon in *N. crassa* [23]. These data indicated that β-glucosidase encoded by *bgl1* was the dominant intracellular enzyme catalyzing the conversion of cellobiose to glucose. Of note, secreted protein in the culture of strain JG412*Δbgl1Δbgl2Δbgl3* was approximately fivefold that observed in parent strain JG412. However, the strategies for constructing CBP-enabling microbes are not the same as those for the development of fungal hyper-producers of cellulolytic enzymes [37]. When converting plant biomass into biochemicals by CBP-enabling microbes, the balance of cellulase secretion for lignocellulose degradation and rapid carbon utilization in the cell is required for elevated yield of the target product. Excessive secretion of protein would increase ATP consumption, which would compromise synthesis of the target product. Only strain JG207*Δbgl2Δbgl3* showed a slightly improved protein secretion and a significantly increased titer of malic acid when grown on cellobiose and Avicel. Further fine tuning of the hydrolytic and phosphorolytic pathways of intracellular cellobiose degradation through improved balance of energy considerations and protein secretion is the next step to improving the performance of *M. thermophila* as a cell factory.

## Conclusions

In this study, we demonstrated that both cellobiose cleavage pathways are used for intracellular cellobiose cleavage in *M. thermophila*. The bioenergetic advantages of phosphorolytic cleavage of cellobiose are propitious to efficient production of malic acid by this fungal CBP system. *Myceliophthora thermophila* displayed divergent regulation patterns of intracellular cellobiose catabolic pathways when grown on cellobiose and cellulose. The hydrolytic pathway plays a major role under cellulose conditions whereas the phosphorolytic pathway contributed more in terms of intracellular cellobiose metabolism under cellobiose conditions. After engineering the two cellobiose metabolic pathways simultaneously, production of malic acid by final strain JG412*Δbgl2Δbgl3* showed significant improvement, producing up to 101.2 g/L and 77.4 g/L malic acid from cellobiose and Avicel, respectively.

## Materials and methods

### Strains and culture conditions

*Myceliophthora thermophila* JG207 was constructed previously, by overexpressing malate transporter gene and pyruvate carboxylase gene in *M. thermophila* ATCC42464 [7]. The JG207 strain and its derivates were grown on 1 × Vogel’s minimal medium supplemented with 2% glucose (MM medium) at 35 °C to obtain conidia, and antibiotic was added when needed for transformant screening.

*Escherichia coli* DH5α was used for vector construction and propagation. Strains were cultivated in Luria–Bertani (LB) medium with 100 µg/mL ampicillin for plasmid selection.

### Vector construction for genetic engineering

For the construction of target genes overexpressing plasmids, cellobiose transporter gene (*cdt*-*1*, NCU00801) amplified from *Neurospora crassa* genomic DNA was ligated between *Spe*I/*Bam*HI of pAN52-PgpdA-bar plasmid carrying the *bar* selectable marker to form *cdt*-*1* overexpressing plasmid P*gpdA*-cdt1-*bar*, using the NEB Gibson assembly kit. The strong constitutive promoter of *eif* (Mycth_2297659) was employed to efficiently overexpress cellobiose phosphorylase genes. The polymerase chain reaction (PCR) fragment of cellobiose phosphorylase gene (*Mtcpp*, Mycth_2308030) from *M. thermophila* genome was amplified using paired primers (Additional file 1). *Ctcpp* (GenBank No. AB013109) from *Clostridium thermocellum* was codon-optimized on the basis of *N. crassa* codon frequency (http://www.kazusa.or.jp/codon/) and artificially synthesized. Terminator Tcbh of *cbh1* (MYCTH_109566) was amplified from *M. thermophila* genome. With the aid of the NEB Gibson assembly kit, the amplicons were ligated between *Bgl*II/*Bam*HI of pAN52-PgpdA-bar plasmid to generate the corresponding plasmids P*eif*-Mtcpp-*bar* and P*eif*-Ctcpp-*bar*.

The construction of sgRNA expression plasmids was performed as described previously [26]. Briefly, using *M. thermophila* genome sequence and the target gene as inputs, the sgRNACas9 tool [38] was used to identify specific sgRNAs target sites in *Mtcpp* (Mycth_2308030), *bgl1* (Mycth_115968), *bgl2* (Mycth_62925), and *bgl3* (Mycth_ 8200). The oligos with low off-target probability was selected and protospacer sequences are presented in Additional file 1. *Myceliophthora thermophila* U6 promoter and a target-directed sgRNA fragment were amplified from U6p-sgRNA plasmid [26], assembled by overlapping PCR, and cloned into a pJET1.2/blunt cloning vector, forming the plasmids U6-*Mtcpp* -sgRNA, U6-*bgl1*-sgRNA, U6-*bgl2*-sgRNA, and U6-*bgl3*-sgRNA.

The vector carrying donor DNA was constructed to perform genomic modification. The 5’- and 3’-flanking fragments of *Mtcpp*, *bgl1*, *bgl2*, and *bgl3* were amplified from the *M. thermophila* genome. These fragments and selectable marker cassettes P*trpC*-*neo* from plasmid p0380-neo [39] were assembled using the NEB Gibson assembly kit and cloned into pPK2BarGFPD and digested with *Spe*I/*Eco*RV to generate the donor DNA sequences donor-*Mtcpp*-neo, donor-*bgl1*-neo, donor-*bgl2*-neo, and donor-*bgl3*-neo.

All vectors were constructed using *E. coli* DH5α and the target genes cloned into shuttle vectors were sequenced to verify the authenticity of the plasmid construction.

### Transformation of *Myceliophthora* protoplasts

Polyethylene glycol-mediated transformation of *M. thermophila* protoplasts was performed as described previously [40]. For gene overexpression, 10 µg of linearized plasmid was transformed into *M. thermophila* protoplasts as needed. A plate supplemented with 100 μg mL^−1^ phosphinothricin was used for transformant selection.

For *Mtccp* deletion, the mixture of PCR amplicons of P*tef1*-*Cas9*-T*tprC* cassette, U6p-*Mtcpp*-sgRNA cassette, and donor-*Mtcpp*-*neo* cassette was co-transformed into *M. thermophlia* JG207 protoplasts.

For multiple gene replacement involving β-glucosidase genes (*bgl1*, *bgl2*, and *bgl3*), sgRNA and donor expression cassettes were mixed with cas9-expression PCR cassette and co-transformed into JG412 strain. The putative transformants were selected with 100 μg/L G418, followed by sequential identification via PCR.

All primer sequences used in this study are listed in Additional file 1.

### Shake flask cultivation

To evaluate the capabilities of malic acid production, batch cultivation was performed in 50 mL of medium inoculated with mature spores to a final concentration of 2.5 × 10^5^ spores/mL in a 250-mL Erlenmeyer flask. The culture was incubated at 45 °C with shaking at 150 rpm in a rotary shaker. Samples (1 mL) were taken at different intervals. Each liter of the cultivation medium contained 75 g of carbon source (cellobiose or Avicel), 0.15 g of KH_2_PO_4_, 0.15 g of K_2_HPO_4_, 0.1 g of MgSO_4_·7H_2_O, 0.1 g of CaCl_2_·2H_2_O, 8 g of Bacto peptone, 1 mL of biotin (0.1 g/L), and 1 mL of trace element of Vogel’s salt, and was sterilized by autoclaving. Subsequently, sterilized CaCO_3_ was added as neutralizing agent to a final concentration of 80 g/L to keep the pH at approximately 6.0.

For intracellular ATP assays and RNA extraction cellobiose or Avicel, the strains were incubated in 50 mL of medium with a final concentration of 40 g/L CaCO_3_ in 250-mL Erlenmeyer flasks at 150 rpm in an orbital shaker for 2 days.

### Metabolite analysis

Prior to organic acid detection in culture broth, 1 mL of 2 M sulfuric acid was added into 1 mL of well-mixed sample in a 15-mL tube and the mixture was incubated at 80 °C for 30 min. The mixture was vortexed at intervals to resolve the dicarboxylic acid adequately. Then, 2 mL of distilled water was added and an aliquot was used for metabolite analysis after mixing. Organic acid was monitored by high-performance liquid chromatography (HPLC) equipped with a Waters 2489 UV detector and an Aminex HPX-87H column (Bio-Rad) at 35 °C. The mobile phase was 5 mM H_2_SO_4_ with a constant flow rate of 0.5 mL/min. Data analysis was performed using the Waters e2695 separation module.

### Assay of mycelium dry weight in culture

Quantification of cell mass was performed after 8 days culture, using a previously described method [41]. Briefly, 2 M HCl was added to a sample of known volume to solubilize undissolved CaCO_3_. The treated broth was centrifuged and washed twice with sterilized water, dried and weighed (w1 = cellulose + mycelium). The residual Avicel (w2 = cellulose) could be measured after solubilizing fungal biomass from the culture with a mixture of acetic acid and nitrate reagent [42], and then the mycelium dry weight could be calculated by subtracting w2 from w1.

### Protein and enzyme activity assay

The assay of secreted protein and enzyme activities in the supernatants of the culture after 4 days on Avicel or cellobiose. Total secreted protein in supernatants was determined using a Bio-Rad protein assay (Bio-Rad) with bovine serum albumin as the standard at 595 nm. Endoglucanase activities of cell cultures were determined by Remazol brilliant Blue R-conjugated CMC purchased from Megazyme. β-Glucosidase activity was assayed with 1.0 mg/mL *p*-nitrophenyl β-d-glucopyranoside (Sigma-Aldrich) as the substrate in 50 mM citrate buffer (pH 4.8) at 50 °C. Sodium carbonate (1 M) was used to terminate the enzymatic reaction after 10 min and the released *p*-nitrophenol (pNP) was measured at 420 nm. One unit (U) of β-glucosidase activity was defined as the number of micromoles of *p*NP released per minute by the enzyme in 1 mL of culture supernatant.

### Cellobiose consumption assays in *M. thermophila*

After 18 h of growth in 100 mL of 1 × Vogel’s salts plus 2% (w/v) glucose at 45 °C, the mycelia were then washed three times in 1 × Vogel’s salts without added carbon and then transferred to Vogel’s salts containing 0.5% (w/v) cellobiose for induction. After an additional 4 h, the mycelia were washed again as above and resuspended in the uptake buffer (1 × Vogel’s salts plus 10 mM cellobiose and 10 μg/mL cycloheximide) for 20 min. The amount of sugar remaining in the supernatant was then determined. After the consumption assay, the fungal biomass was blotted dry and completely dried overnight at 105 °C to determine the dry weight for data normalization.

### Quantitative real time-PCR analysis

For the assay of relative transcription levels of targe genes, sample preparation and RNA extraction were performed using the method described previously [7]. Quantitative PCR was carried out with SYBR Green Realtime PCR Master Mix (Toyobo, Osaka, Japan) using a CFX96 real-time PCR detection system (Bio-Rad). The PCR reaction mixture (with three replicates) included 75 ng of template RNA, 0.4 μL of each primer (10 μM), 10 μL of RNA-direct SYBR^®^ Green Realtime PCR Master Mix, and 8.2 μL of H_2_O. Negative controls contained an equal volume of water instead of RNA. Actin gene (MYCTH_2314852) was used as an internal control. The relative transcript level of each gene was calculated by the 2^−ΔΔCt^ method.

For copy number assay of genes ectopically inserted into *M. thermophila* genome, fungal genomic DNA was extracted from transformants as described previously and used as the template for RT-qPCR. Quantitative PCR was carried out with SYBR Green Realtime PCR Master Mix (Toyobo, Osaka, Japan) with a CFX96 real-time PCR detection system (Bio-Rad), according to the manufacturer’s instructions. The oligonucleotides of the primers for each gene were optimized to obtain amplification efficiency between 95 and 105% and only one melting temperature on the melting curve.

The primers used for RT-qPCR are listed in Additional file 1.

### Measurement of intracellular ATP levels and cellobiose phosphorylase activity

A 50-mL sample of mycelial medium was poured into a Buchner funnel fitted with four pieces of gauze. The residue was washed with distilled water until most CaCO_3_ was removed, and then immediately homogenized in liquid nitrogen and stored at − 80 °C. A prechilled mortar and pestle were used for frozen mycelia disruption. The resulting paste was transferred into l mL of phosphate-buffered saline (pH 7.4). After centrifugation for 10 min at 4 °C, clear supernatant was used for protein quantitation and further assay.

The intracellular ATP concentration was determined using the ATP Quantification kit (Sigma-Aldrich) according to the manufacturer’s instructions and normalized to the protein concentration in the extract.

The measurement of cellobiose phosphorylase activity was performed at 45 °C in an assay mixture (100 μL) consisting of 50 mM phosphate buffer (pH 7.4) and 10 mM cellobiose for 10 min. The reaction was stopped completely by adding 50 μL of Tris–HCl buffer (4 M, pH 7.0). Glucose-1-phophate concentrations were determined continuously using the glucose-1-phophate Assay Kit (Sigma-Aldrich) according to the provided protocol. One unit of cellobiose phosphorylase activity was defined as the amount of cellobiose phosphorylase releasing 1 μmol of glucose-1-phophate from cellobiose per minute.

### Statistical significance tests

A one-tailed homoscedastic (equal variance) *t* test was employed for all statistical significance tests, unless otherwise noted. n.s represented *p*-value > 0.05; * represented *p*-value < 0.05; ** represented *p*-value < 0.01 and *** represented *p*-value < 0.001.

## Supplementary information

**Additional file 1.** Primers used for the genetic manipulation in *M. thermophila.*

**Additional file 2.** PCR analysis of the mutants of *M. thermophila* generated in this study.

**Additional file 3.** Dry cell weigh of in the culture of the strain strain JG207*ΔMtcpp* grown on cellobiose and Avicel for 8 days.

**Additional file 4.** Copy number assay by RT-qPCR. *cdt*-*1* in strain JG207cdt; *Ctcpp* in strain JG412; *Mtcpp* in strain JG413.

## Data Availability

All data generated or analyzed during this study are included in this published article and its additional files.

## Abbreviations

ATP: adenosine triphosphate
Avicel: microcrystalline cellulose
BG: β-glucosidase
Cas9: CRISPR associated protein 9
CBP: consolidated bioprocessing
CDT: cellodextrin transporter
CPP: cellobiose phosphorylase
CRISPR: clustered regularly interspaced short palindromic repeats
HXK: hexose kinase
PCR: polymerase chain reaction
RT-qPCR: quantitative real time polymerase chain reaction
rTCA: reductive tricarboxylic acid
