# Supplementary material for: Dissecting cellobiose metabolic pathway and its application in biorefinery through consolidated bioprocessing in Myceliophthora thermophila
Source: Fungal Biol Biotechnol. 2019 Nov 13;6:21. doi: 10.1186/s40694-019-0083-8 (PMC6852783; doi:10.1186/s40694-019-0083-8)
Supplement: Supplementary file 1 — Additional file 1. Primers used for the genetic manipulation in M. thermophila. [file 40694_2019_83_MOESM1_ESM.pdf]

Additional file 1. Primers used for the genetic manipulation in *M. thermophila*.

Name	Prime sequence (5’-3’)	Note
**Construction of expression vector**		
Peif-F	GCTGACTTGAAGTAATCTCTGCAGATCTCCACATCATGTAAACAGAGGC	Cloning of Peif
Peif-R	CTTGTTGTTGTTGTTGTGTTG	Cloning of Peif
Tpgk-F	GTCTCGAGAGAGAGAGAGAGTG	Cloning of Tpgk
Tpgk-R	TCCTTCAATATCAGATAACGTCGACTCTAGATCTCCTCGTCATCTCGGTTG	Cloning of Tpgk
cdt-F	CTCCCCACATCACAGAAATCAAAACTAGTATGTCGTCTCACGGCTCCCA	Cloning of *cdt-1*
cdt-R	TTTCAGTAACGTTAAGTGGATCCGAATTCCTAAGCAACGATAGCTTCGGAC	Cloning of *cdt-1*
Ctcpp-F	CAACACAACAACAACAACAAGATGAAGTTCGGGTTTTTCGATG	Cloning of *Ctcpp*
Ctcpp-R	CACTCTCTCTCTCTCTCGAGACTCAGCCCATAATGACCTCGAC	Cloning of *Ctcpp*
Mtcpp-F	CAACACAACAACAACAACAAGATGAGGAAAGAAGGCTCGCT	Cloning of *Mtcpp*
Mtcpp-R	CACTCTCTCTCTCTCTCGAGACCTACTGAGGCACCAGAACCGA	Cloning of *Mtcpp*
**Construction of sgRNA expressing plasmids**		
U6p-F	AGGATCGGTGGAGTGAAGTTCGGAA	Cloning of U6 promotor
U6p-Mtcpp-R	CTAGCTCTAAAACGGTGGTGGGCATAGTAGTTCGAGGAAAGAAAGAAAAGAAG	Cloning of U6 promotor
U6p-bgl1-R	CTCCTCTTCTTTTCTTTCTTTCCTCGACACATTCTGCGCCATCCCGTTTTAGAGC	Cloning of U6 promotor
U6p-bgl2-R	TAGCTCTAAAACTATTCCATGTTGCTGCCAGCGAGGAAAGAAAGAAAAGAAGAG	Cloning of U6 promotor
U6p-bgl3-R	TTCTAGCTCTAAAACCTTAGAATCGCGGAGGAGCCGAGGAAAGAAAGAAAAGAAG	Cloning of U6 promotor
gMtcpp-F	TTTCTTTCTTTCCTCGAACTACTATGCCCACCACCGTTTTAGAGCTAGAAATAGCA	*Mtcpp*-gRNA5’
gbgl1-F	TTCTTTCCTCGACACATTCTGCGCCATCCCGTTTTAGAGCTAGAAATAGCAAGTT	*bgl1*-gRNA5’
gbgl2-F	TTCTTTCCTCGCTGGCAGCAACATGGAATAGTTTTAGAGCTAGAAATAGCA	*bgl2*-gRNA5’
gbgl3-F	TCTTTCTTTCCTCGGCTCCTCCGCGATTCTAAGGTTTTAGAGCTAGAAATAGC	*bgl3*-gRNA5’
gRNA-R	AAAAAGCACCGACTCGGTGCCACTT	gRNA3’
**Donor DNA construction**		
neo-F	CGACGTTAACTGATATTGAAGGA	Cloning of P*trpC-neo*
neo-R	TCAGAAGAACTCGTCAAGAA	Cloning of P*trpC-neo*
Mtcpp-5’-F	AGTGGGCGCTTACACAGTACACGAGGACTTAGCATCAACAAACCAACCCCA	*Mtcpp*-upstream
Mtcpp-5’-R	CCAAAAAAATGCTCCTTCAATATCAGTTAACGTCGTTAAGGTCTTGGCTTCAATGTTG	*Mtcpp*-upstream
Mtcpp-3’-F	ATCGCCTTCTATCGCCTTCTTGACGAGTTCTTCTGATCGTGGGATGCCAATCAACAG	*Mtcpp*-downstream
Mtcpp-3’-R	GTCATGTGATTGTAATCGACCGACGGAATTGAGGATGCCGTCCCAGAGGTACTTGTTG	*Mtcpp*-downstream
bgl1-5’-F	AGATGTGGAGTGGGCGCTTACACAGTACACGAGGACTTCACCCCCAGAATCAGCCGA G	*bgl1*-upstream
bgl1-5’-R	AAATGCTCCTTCAATATCAGTTAACGTCGTGGAGGGGCCACGGCCATCCTCGTT	*bgl1*-upstream
bgl1-3’-F	CTTCTATCGCCTTCTTGACGAGTTCTTCTGATGTTCAAGGCCATCCCCAAGTGCAAG	*bgl1*-downstream
bgl1-3’-R	CAAGTCATGTGATTGTAATCGACCGACGGAATTGAGGATCAGAGACAGGAGCCGTCA	*bgl1*-downstream
bgl2-5’-F	GGAGTGGGCGCTTACACAGTACACGAGGACTTAACCTCTCCGTCTCCTCTGCT	*bgl2*-upstream
bgl2-5’-R	CCCAAAAAAATGCTCCTTCAATATCAGTTAACGTCATACCGCAGGGGAAAAGCGCAG	*bgl2*-upstream
bgl2-3’-F	CATCGCCTTCTATCGCCTTCTTGACGAGTTCTTCTGAGAAGTGGATATGACGGACTG	*bgl2*-downstream
bgl2-3’-R	AGTCATGTGATTGTAATCGACCGACGGAATTGAGGATAACACGACCGGCTGGCCCTC	*bgl2*-downstream
bgl3-5’-F	TGGAGTGGGCGCTTACACAGTACACGAGGACTTCCTTCGCCTGTTGATAACAGG	*bgl3*-upstream
bgl3-5’-R	CAAAAAATGCTCCTTCAATATCAGTTAACGTCGTCAGATGGCTCCCGTCGCGAGCC	*bgl3*-upstream

bgl3-3'-F	GCCTTCTATCGCCTTCTTGACGAGTTCTTCTGAATTGCCAAGACGTGGGCCGAG	*bgl3*-downstream
bgl3-3'-R	CATGTGATTGTAATCGACCGACGGAATTGAGGATCAGCTGGTACCAGGCCGCAAC	*bgl2*-downstream
**PCR analysis of gene disruption**		
Mtcpp-out-F	GAACCAAAAGATGATGATCCAA	Detecting *Mtcpp* disruption
Mtcpp-out-R	CGGCATTAAGCTCGATGCGGTA	Detecting *Mtcpp* disruption
bgl1-out-F	CTCGGCTCATTGGGGCCTACTA	Detecting *bgl1* disruption
bgl1-out-R	CCGGGTACTCGCCAAAGTAGAT	Detecting *bgl1* disruption
bgl2-out-F	ATCCAAGAACATCCCGGCAGTA	Detecting *bgl2* disruption
bgl2-out-R	TGGACGCCGTTGACAAGGTT	Detecting *bgl2* disruption
bgl3-out-F	CACGGTCTCAGCAGTCACGTA	Detecting *bgl3* disruption
bgl3-out-R	TTGCGCCTACCGTGATGCTTG	Detecting *bgl3* disruption
**RT-qPCR analysis**		
actin-F	TGCCCATCTACGAGGGTTTC	Detecting actin gene
actin-R	TTGATGTCACGGACAATTTCAC	Detecting actin gene
qMtcpp-F	AGCGTCTTCTATGTCCACAGC	Detecting *Mtcpp*
qMtcpp-R	CGGTAGTAGTTGGGCAGGAAG	Detecting *Mtcpp*
qBG1-F	AGTTCGCCATCTCGTGGTTCG	Detecting *bgl1*
qBG1-R	CATGCCGTAGAAGTCGTTGGAG	Detecting *bgl1*
qBG2-F	CTGGAACTGGACCGAGGAG	Detecting *bgl2*
qBG2-R	CTGCTTCTGCTTGGCGATC	Detecting *bgl2*
qBG3-F	TGGCGGCAAGGTCATGTAC	Detecting *bgl3*
qBG3-R	GTTGAAGGTGGTGTAACTGAG	Detecting *bgl3*
