# Supplementary material for: Dissecting cellobiose metabolic pathway and its application in biorefinery through consolidated bioprocessing in Myceliophthora thermophila
Source: Fungal Biol Biotechnol. 2019 Nov 13;6:21. doi: 10.1186/s40694-019-0083-8 (PMC6852783; doi:10.1186/s40694-019-0083-8)
Supplement: Supplementary file 2 — Additional file 2. PCR analysis of the mutants of M. thermophila generated in this study. [file 40694_2019_83_MOESM2_ESM.pdf]

Strains	Primers	PCR analysis
JG207ΔMtcpp	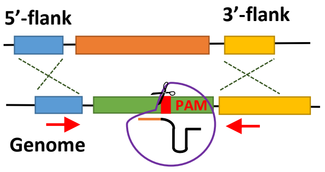	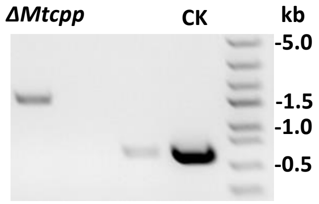
JG207cdt	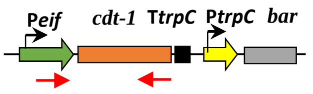	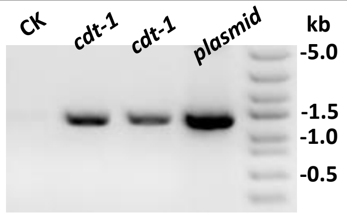
JG413	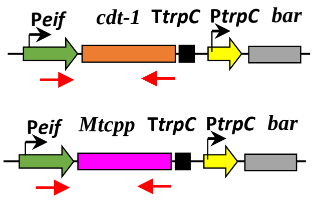	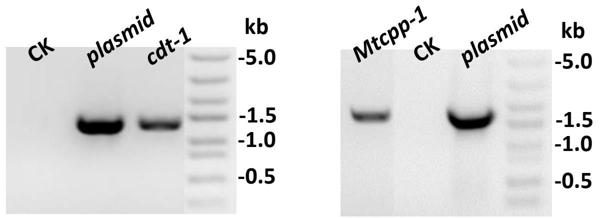
JG412	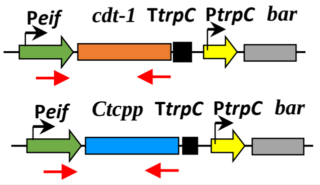	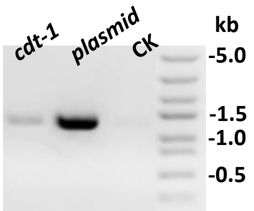
JG412Δbgl1	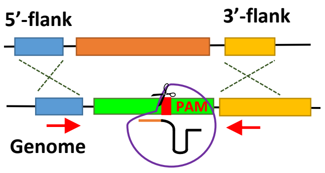	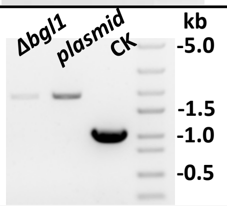
JG412Δbgl2	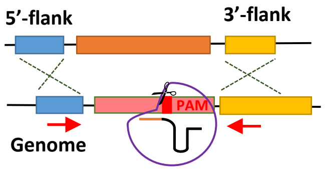	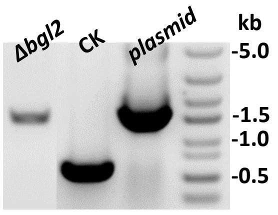
JG412Δbgl3	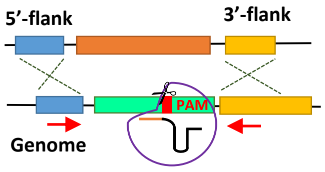	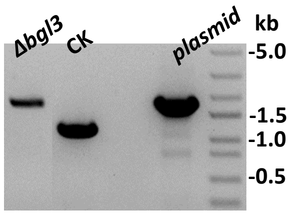
JG412Δbgl1Δbgl2	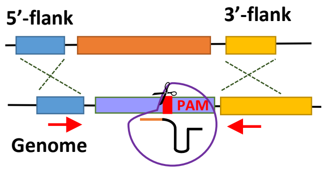	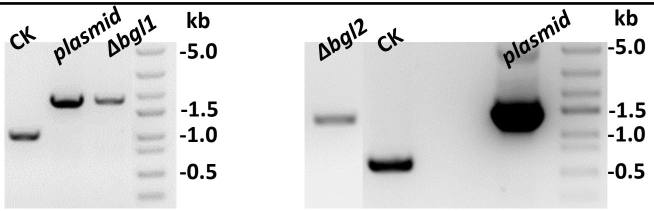
JG412Δbgl1Δbgl3	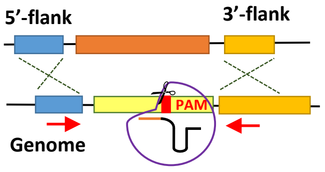	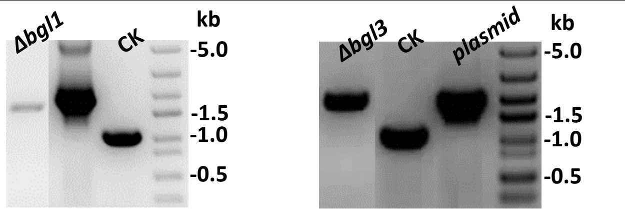
JG412Δbgl2Δbgl3	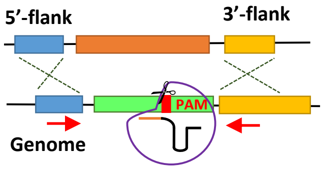	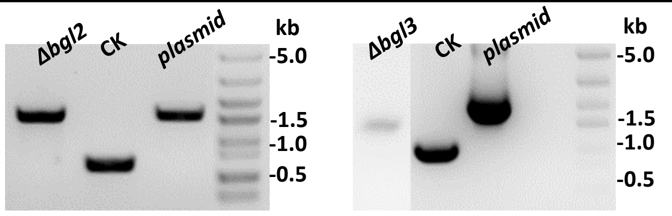
JG412Δbgl1Δbgl2 Δbgl3	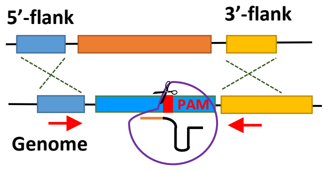	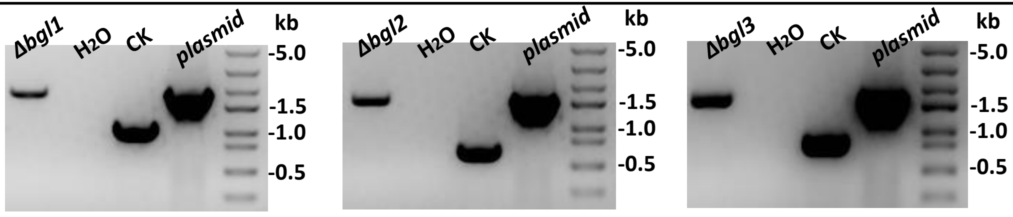
