# Supplementary figures and images for: Dissecting cellobiose metabolic pathway and its application in biorefinery through consolidated bioprocessing in Myceliophthora thermophila
Source: Fungal Biol Biotechnol. 2019 Nov 13;6:21. doi: 10.1186/s40694-019-0083-8 (PMC6852783; doi:10.1186/s40694-019-0083-8)

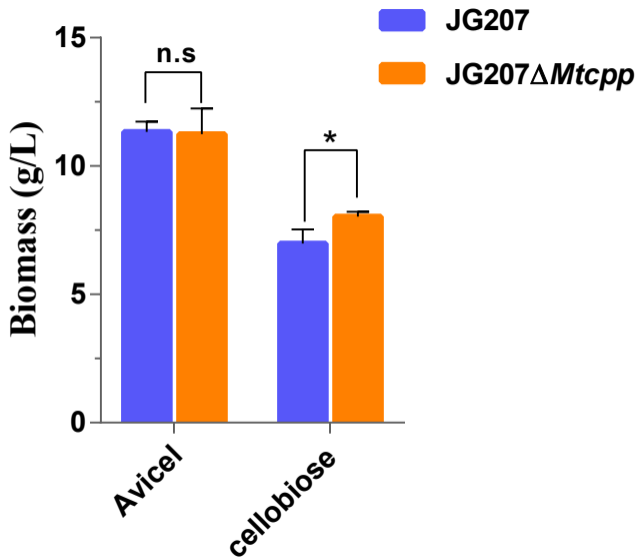

Supplement: Supplementary file 3 — Additional file 3. Dry cell weigh of in the culture of the strain strain JG207ΔMtcpp grown on cellobiose and Avicel for 8 days. [file 40694_2019_83_MOESM3_ESM.pdf]

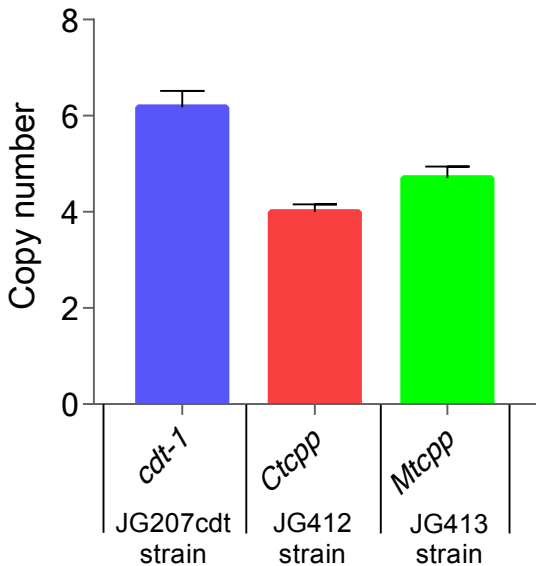

Supplement: Supplementary file 4 — Additional file 4. Copy number assay by RT-qPCR. cdt-1 in strain JG207cdt; Ctcpp in strain JG412; Mtcpp in strain JG413. [file 40694_2019_83_MOESM4_ESM.pdf]
